# A Promise of Dawn

**Published:** 1890-01-04

**Authors:** 


### The Hospital

A Weekly Journal of

**Science, Medicine, Nursing, and Philanthropy.**

*For Hospitals, Asylums, Medical Practitioners, Students, Nurses, Families, Parishes, Congregations, and the Charitable Public.*

Vol. VII. No. 171.] [January 4, 1890.

### A Promise of Dawn.

"I never knew any man in my life who could not bear another's misfortunes perfectly like a Christian." Thus wrote Pope! "Hobbes clearly proves that every creature lives in a state of war by nature." Thus Swift! "Persuasion is the resource of the feeble: but the feeble can seldom persuade." Thus Gibbon. In the face of the very considerable pessimism of many of the thoughtful of to-day, and of the cynical scepticism about human nature which characterised so many of the wits and writers of the time preceding our own, it seems an adventure of some boldness to try to prove that we are approaching the dawn of a better day. Nevertheless, it is our emphatic conviction that in the English-speaking world selfishness is diminishing, intelligence is increasing, and a kindly interest in the welfare of others is becoming a *sine qua non* of good manners and gentle breeding.

It is possible to observe life and manners from several standpoints. The usual course is for each man to be content to regard it solely from his own. Thus, to the doctor the whole population of the world is apt to consist of patients, whose cases are of varying degrees of interest and whose powers of paying fees are greater or less. The lawyer, on his part, sees a possible litigant in every man he meets on the highway; whilst the Member of Parliament, conscious of uncertain merit, encounters nothing but doubtful voters in his daily walks. But if a man really wishes to take a just measure of his own times he must detach himself entirely from the obscuring circumstances of his own particular business and look squarely on the world, without prejudice, without passion, and in the light that shines from comparison with other times. If the writers of the eighteenth century, and the historians who record its doings and its thinkings, do not lie, then the present century, as compared with the last, is, in gentle manners, diffused intelligence, and kindly feeling, what the soft radiance of advancing dawn is to the blackness that enshrouds the midnight hour.

We have no wish to make our own time appear either better or worse than it really is. Our purpose in this journal is always and everywhere practical, and practical purposes are never permanently advanced by distorted facts or special pleadings. But this convincing proof of improved morals presents itself to the mind—namely, that in addressing a philanthropic argument to readers of the present day we are able to take higher ground, to appeal to worthier motives, and to feel confident of larger and more generous results than could have been the case with anyone called upon to address an indiscriminate audience of the contemporaries of Mr. Pope. There is every reason to believe that a large majority of our readers are not only willing to be approached on philanthropic subjects, but are anxious to meet us half-way; are not only willing to be convinced by arguments, but are grateful to be shown where duty lies and to be encouraged in the doing of it. This is a totally different state of public feeling from what philanthropists had to contend with in the days of Howard and of those who went before him. So much, at least, everybody will admit, and so much all wholesome-minded men are profoundly thankful for.

Practical philanthropy is a business which can never be retired from. He that once puts his hand to this plough cannot look back without moral injury and loss. If his resources be diminished for any reason, it may be necessary for him to curtail his benevolent operations. But that is not the same as the weakening of his benevolent purposes and the loss of his generous impulses. A man may be so reduced that he shall not have even a penny to give to the shivering crossing sweeper on a wintry night, and yet his soul may be as full of divine charity as that of a Shaftesbury, a Wilberforce, or a Fry. It is with the consciousness that we are addressing those who make the promotion of the well-being and happiness of their fellow-men a serious duty that we offer to them a few special words at the beginning of the New Year.

The hospital manager, in considering the prospects of his institution for the coming year, cannot but note several circumstances of a favourable and encouraging kind. Among these, one of the chief is the undoubted increase of the general prosperity of the country. Trade has revived, profits have increased, and people of all classes are almost universally employed. Those who have always given can now give more freely, and those who have never given at all can afford to begin. We do not think it is either wise or just to raise a menacing finger against the men and women who have nobly stuck to the ship of charity through distress and storms. To sternly reprove those who already give something because they do not give more savours of pharisaism and an unappreciative spirit. To them we offer a kindly suggestion that the hospitals can do more work if they are supplied with more means, and that more work is constantly requiring to be done.

The case of those who have never supported hospitals at all is different. From any point of view, either of duty or of self-interest, it cannot be questioned that the prosperous ought to help the unprosperous. Every human being has a well-defined claim on every other human being. More especially has every Briton a claim upon every other Briton, and, most of all, has every immediate neighbour a claim upon every other neighbour. The creature who doubts this is base, if he be not vicious. According to the standard of Darwin he is of low intellectual and moral development; according to the standard of the New Testament he is selfish, wicked, and worthless. To him, therefore, we say with all possible boldness, that he needs to reconsider his position, and to take prompt steps to place himself on a level with those who are worthy to bear the honourable and honoured name of "man."

Not only are the prosperous times favourable to hospitals, but the administrative record of those institutions during the past few years has steadily improved. Of this there cannot be the slightest doubt. At several hospitals of the most unsatisfactory repute full and searching enquiries have recently been publicly made. Those enquiries, whilst not always improving the reputation of particular individuals, have nevertheless demonstrated this all-important fact, that such is the degree of care exercised in the management of the most faulty institutions that serious malversation is practically impossible. It is not said that hospital administration, even in the highest quarters, approaches a standard that is faultless. On the contrary, much remains to be done before perfection can be brought even within sight. But this, at least, every reader may take absolutely for granted, that the money spent at hospitals is spent by managers of intelligence and integrity, who devote a very great deal of valuable time to their onerous work, and whose one purpose it is to render the worthiest possible account of the trust committed to their charge.

Several circumstances have conspired to improve the management of hospitals in recent years. The Hospitals Association, consisting of persons of high position and proved experience, has devoted itself with both zeal and energy to the practical problems of administration. A great deal of light has thus been thrown on hospital affairs, abuses and extravagances have been put to shame, and there has been a general tightening of the cords of wholesome discipline and a marked elevation of the standard of official opinion and practice. This journal, we are happy to think, has had no small share in the good work which has been effected. It has been, at least our intention, to do justice both to subscribers and to officials, both to doctors and to patients. We have no wish to claim for ourselves the smallest modicum of judicial infallibility. Our general purpose is far otherwise than that. We have striven to avoid small and carping criticism; to extenuate, rather than to drag to light unimportant and unintentional faults; and to deal candidly and honestly, but without passion, with those who, either through ignorance or wilfulness, have failed to show any true appreciation of the claims of public duty. Whatever may have been our successes, these have been our aims; and we are not without good ground for believing that we have helped in some measure both to improve the administration of hospitals and to strengthen public confidence in their worth. One last word we may be permitted to say, and it is this, that if ever hospitals as a whole deserved the confidence and support of a generous-hearted public, it is now.

